# A Peculiar Phenomenon in Cerebral, and Especially in Meningitic, Affections

**Published:** 1886-02

**Authors:** 


### A Peculiar Phenomenon in Cerebral, and especially in Meningitic, Affections.

While presenting a meningitic patient to the class on the 1st of December, Prof. Henoch, of the Berlin University, called attention to a peculiar symptom almost invariably observable in cerebral, and especially in meningitic, affections, which, on account of its diagnostic value, deserves to be remembered. The patient, on being examined, did not present any muscular contraction in either the legs or arms, but if the thigh was flexed at right angles to the abdomen, great muscular rigidity and contractions at once set in, which prevented the part from being extended. The same phenomenon, though not so conspicuously, could be noted in the arm. This peculiar condition, Henoch said, pointed invariably to a cerebral affection, and possessed an especial value if the meningitic lesions were in the initiatory stage, and presented few or no other symptoms.

Examining the literature of this noteworthy phenomenon, we find in the *Berl. Klinische Wochenschrift* of November 23, 1885, that Dr. W. Kernig, of St. Petersburg, was the first to describe this symptom, and to attempt to furnish a rational explanation of it. This author observed the stated symptom in fifteen cases of inflammation of the pia mater, thirteen cases of which were epidemic cerebro-spinal meningitis, one a tubercular and one a purulent meningitis. Besides, he noted the same occurrence in six other cases, viz.: bloody extravasations of the cerebrum and the meningeal membranes, pachy- and lepto-meningitis, after thrombosis of the tranverse sinus. The symptom appeared in the beginning of the several affections, lasted during the entire course of the disease, and was even demonstrable after reconvalescence had set in.

We likewise find that Dr. Ed. Bull, of Christiania, observed this contraction symptom in three cases, one of which was a tubercular meningitis, the second a cerebral tumor, and the third a thrombosis of the tranverse sinus.

An explanation of this singular phenomenon appears not easy, though it is evident that the contraction must be classed among the neuropathic spastic appearances. Its origin seems central, though a peripheral reflex influence may also participate in its causative agencies.

Examining in normal individuals the extended knee-joint, if the hip-joint is kept at the same time in a flexed position, we find that a perfect flexion of the knee is usually only possible if the angle between thigh and abdomen amounts to but little less than 90°. If, on the contrary, the thigh is flexed strongly towards the abdomen, forming an acute angle, the knee-joint cannot be made to form an angle of 180°; the knee forms an obtuse angle, and a further flexion is prevented by the tension of the muscles on the back of the thigh. These remarks are not superfluous, as they show that under normal conditions a similar contraction occurs, which, of course, ought not to be mistaken for a symptom of meningitis. To avoid errors, it is best to bend the knee-joint at an angle of 90°, which is most appropriately effected by making the patient sit on the margin of the bed.—*Therapeutic Gazette.*

